# *Helicobacter pylori* Infection Synergizes with Three Inflammation-Related Genetic Variants in the GWASs to Increase Risk of Gastric Cancer in a Chinese Population

**DOI:** 10.1371/journal.pone.0074976

**Published:** 2013-09-19

**Authors:** Miao Li, Liu Huang, Hong Qiu, Qiang Fu, Wen Li, Qianqian Yu, Li Sun, Lihong Zhang, Guangyuan Hu, Junbo Hu, Xianglin Yuan

**Affiliations:** 1 Department of Oncology, Tongji Hospital, Huazhong University of Science and Technology, Wuhan, Hubei Province, China; 2 Department of Surgery, Tongji Hospital, Huazhong University of Science and Technology, Wuhan, Hubei Province, China; The University of Texas MD Anderson Cancer Center, United States of America

## Abstract

**Background:**

Three recent genome-wide association studies (GWASs) have reported that three SNPs (rs4072037, rs13361707 and rs2274223) located on genes related to host inflammatory response are significantly associated with susceptibility to gastric cancer (GC) in Chinese populations. *Helicobacter pylori* infection is also an important risk factor for GC through causing inflammatory response in the gastric mucosa. However, no study has established whether there are potential gene-environment interactions between these genetic variants and *H. pylori* infection to the risk of GC.

**Methods:**

We genotyped three polymorphisms (rs4072037 at 1q22, rs13361707 at 5p13, and rs2274223 at 10q23) in 335 Chinese gastric adenocarcinoma patients and 334 controls. *H. pylori* serology was examined by enzyme-linked immunosorbent assay. Multivariable logistic regression models were used to evaluate the association between the variables and GC risk.

**Results:**

We confirmed that the three SNPs (rs4072037, rs13361707 and rs2274223) were significantly associated with GC susceptibility. *H. pylori* infection also significantly increased the risk of GC. Furthermore, there were joint effects between *H. pylori* infection and the three SNPs on the risk of GC. The most elevated risk of GC was found in subjects with *H. pylori* seropositivity and AA genotypes for rs4072037 [odds ratio (OR), 3.95; 95% confidence interval (CI), 2.29–6.79], *H. pylori* seropositivity and CT/CC genotypes for rs13361707 (OR, 2.68; 95% CI, 1.62–4.43), *H. pylori* seropositivity and AG/GG genotypes for rs2274223 (OR, 2.45; 95% CI, 1.55–3.88) compared with those with *H. pylori* seronegativity and other genotypes of each SNP. Significant interactions were observed between *H. pylori* seropositivity and the three SNPs (all *P*_G× E_ <0.05) to the risk of GC.

**Conclusion:**

These findings indicate that the three SNPs (rs4072037, rs13361707 and rs2274223) identified in the GWASs may interact with *H. pylori* infection to increase the risk of GC.

## Introduction

Gastric cancer (GC) is a worldwide disease and the second most common cause of cancer-related deaths [1]. The incidence of GC varies geographically and 70% of new cases and deaths occur in developing countries. In China, 0.4 million new cases and 0.3 million deaths were estimated for GC in 2005 [2]; therefore, preventing and controlling this malignancy remains a key public health issue. It is widely accepted that gastric carcinogenesis is a multifactorial process that is related to lifestyle (e.g. smoking, low vegetable/fruit consumption, and high salt/nitrates intake), socioeconomic status, pernicious anemia, *Helicobacter pylori* infection, and host genetic polymorphisms [3,4].

*H. pylori* infection is a well-established risk factor in gastric carcinogenesis and is classified as a Class I human carcinogen by the World Health Organization (WHO) based on epidemiological evidence [5]. The prevalence of *H. pylori* is higher in developing than developed countries [6]. *H. pylori* can adhere to host gastric mucosa and cause transition from normal mucosa to chronic superficial gastritis, which then increases the risk of atrophic gastritis and intestinal metaplasia, and eventually leads to the development of GC in some of the infected individuals over many years [4]. Although GC is associated with *H. pylori* infection in the stomach, only a small proportion of exposed individuals develop this common malignancy in their lifetime, indicating the important roles of the host genetic factors in the etiology of GC.

Recently, three genome-wide association studies (GWASs) related to GC in the Chinese population have been published [7–9]. These studies identified some new genetic susceptibility loci for GC in a large sample of the Chinese population. These susceptibility loci have subsequently become a new focus of research, and important in understanding the pathogenesis and prognosis of GC [10,11]. In the current study, we were particularly interested in the three GWAS-identified single nucleotide polymorphisms (SNPs) that are located on genes related to host inflammatory responses. Among them, rs4072037 at 1q22 is a synonymous SNP in the second exon of mucin1 (MUC1), which is associated with attenuation of intracellular levels of reactive oxygen species (ROS), as well as the epithelial infectious and inflammatory diseases [12]. Rs13361707 is the index SNP among several across the 5p13.1 region that are associated with GC susceptibility in the GWAS [9], and it is located close to the gene for prostaglandin E receptor 4 (PTGER4). PTGER4 is a prostaglandin (PG) E2 receptor that is the major product of cyclooxygenase (COX)-2, which plays an important role in the immune response. Besides, rs2274223 is localized to the 26th exon of phospholipase C ε1 (PLCE1), a member of the PLC protein family, which is related to expression of inflammation factors in tumor-associated inflammation [13].

It has been increasingly accepted that the etiology and mechanism of GC involve not only genetics or environmental factors alone, but also interactions between them [14]. Many studies have shown that *H. pylori* infection is associated with polymorphisms of some inflammatory factors to affect the risk of GC [15,16]. However, to the best of our knowledge, no studies have investigated the potential gene-environment interactions between *H. pylori* infection and the above genetic loci to the risk of GC. The current independent case–control study with 335 GC cases and 334 controls in a Chinese population was conducted to investigate the possible interactions between the three SNPs (rs4072037, rs13361707 and rs2274223) and *H. pylori* infection to the risk of GC.

## Materials and Methods

### Study population and data collection

All study participants provided written informed consent before blood samples were collected. The study was approved by the Ethics Committee of Tongji Medical College. A total of 335 GC patients were consecutively enrolled from Tongji Hospital of Huazhong University of Science and Technology from February 2011 to August 2012. All cases were newly diagnosed and histopathologically confirmed gastric adenocarcinoma, without a previous malignant tumor in any other organ or any antitumor therapy before blood sample collection. The tumor-node-metastasis (TNM) stages were evaluated according to the American Joint Committee on Cancer Cancer Staging Manual, 6th edition. Additionally, patients who had received any blood transfusion during the past 6 months or who were receiving immunosuppressive therapy were excluded. Three hundred and thirty-four cancer-free controls were randomly selected from individuals who were undergoing health examination at the same hospital at the same time. The inclusion criteria for controls were absence of history of cancer and frequency-matching to the cases by age (±5 years) and sex. All of the cases and controls were genetically unrelated and limited to Han Chinese ethnicity. Demographic and epidemiological information including age, sex, ethnicity, body mass index (BMI, calculated from the body weight in kilogram and height in meters according to the formula: kg/m^2^), smoking and alcohol status, medical history and family history of cancer were recorded on a questionnaire through face-to-face interviews by a trained doctor or medical student. After recruitment, 5 ml peripheral venous blood was obtained from each participant.

### Selection of SNPs and Genotyping

We used the National Center for Biotechnology Information (NCBI)’s Pubmed database (http://www.ncbi.nlm.nih.gov/pubmed/). There are many newly identified susceptibility loci in recently published GWASs of GC in China [7–9]. We only selected SNPs located on genes related to host inflammatory responses. As five SNPs on the gene PLCE1 at 10q23 had strong pair-wise linkage disequilibrium (LD) in the GWAS [7], we chose the most significant nonsynonymous variants. Therefore, three such SNPs, rs4072037 at 1q22, rs13361707 at 5p13 and rs2274223 at 10q23 were included in this study. Genomic DNA was extracted from peripheral blood using a Fuji whole blood DNA kit (Fujifilm Corporation, Tokyo, Japan) following the manufacturer’s instructions and stored at -80°C until use. Three SNPs (rs4072037, rs13361707 and rs2274223) were all genotyped using TaqMan assay with ABI 7900HT real-time PCR System (Applied Biosystems, Foster City, CA, USA) as previously described [17]. The call rate for each SNP was >95%. The genotyping results were analyzed using SDS 2.3 Allelic Discrimination Software (Applied Biosystems). Ten percent of the samples were randomly selected for repeat genotyping and the results were 100% concordant.

### *H. pylori* serological testing

We tested for *H. pylori* infection using an *H. pylori* IgG enzyme-linked immunosorbent assay (ELISA) kit (Shenzhen Yahuilong Biotech Company, Shenzhen, China) according to the manufacturer’s instructions. Samples with optical density (OD) readings higher than the threshold value of 10AU/ml were considered to have *H. pylori* seropositivity. We also randomly chose 10% of the samples and obtained 95.5% concordance on the repeat assays.

### Statistical analysis

A χ^2^ test was used to examine the distribution of demographic characteristics among the GC and control groups in terms of sex, age, BMI, cigarette smoking, alcohol consumption, and *H. pylori* infection. Hardy–Weinberg equilibrium for each SNP was assessed using the goodness-of-fit χ^2^ test among the controls. Logistic regression was used to analyze adjusted odds ratios (ORs) and 95% confidence intervals (CIs) of the studied SNPs, individually or in combination, for the risk of GC. Gene × environment interaction was calculated by conducting a 1-degree-of-freedom Wald test of a single interaction parameter (gene × *H. pylori* infection) in logistic regression with age, sex, BMI, smoking and drinking status as covariates as previously described elsewhere [18]. Association analysis of *H. pylori* serology and genotypes was further stratified by TNM tumor stage. Benjamini and Hochberg’s method was used to adjust *P* values in multiple testing to minimize the possibility of type 1 error. All data analyses were performed with SPSS for Windows version 17.0 (SPSS Inc., Chicago, IL, USA). All statistical tests were two-sided, and *P*<0.05 was accepted as statistically significant.

## Results

### Characteristics of study subjects

The frequency distribution of sex, age, BMI, cigarette smoking, alcohol consumption, *H. pylori* infection, and clinical features between the GC cases and controls is summarized in Table **1**. Two characteristics, smoking status and *H. pylori* infection, were significantly more common in cases with GC than in controls (*P* = 0.002 for smoking status and *P* = 0.0003 for *H. pylori* infection). There were no significant differences in the distribution of age, sex, BMI, and drinking status between cases and controls. Of the 335 GC cases, 78 (23.3%) were classified as cardia GC and 257 (76.7%) as non-cardia GC. Furthermore, 96 cases (28.7%) presented with stage I/II and 239 (71.3%) with stage III/IV disease.

**Table 1:** Distribution of selected variables in patients with GC and cancer-free controls.

**Variables**		**Cases (*n* = 335**)			**Controls (*n* = 334**)		***P* value^a^**
		***n***	**%**		***n***	**%**	
**Age (years)**							0.575
<40		42	12.5		34	10.2	
40–60		204	60.9		204	61.1	
>60		89	26.6		96	28.7	
**Sex**							0.660
Male		230	68.7		224	67.1	
Female		105	31.3		110	32.9	
**Body mass index (Kg/m^2^)**							0.806
<18.5		14	4.2		11	3.3	
18.5–23.9		166	49.6		175	52.4	
≥24		155	46.3		148	44.3	
**Smoking status**							0.002
Never		187	55.8		226	67.7	
Ever		148	44.2		108	32.3	
**Drinking status**							0.450
Never		214	63.9		223	66.8	
Ever		121	36.1		111	33.2	
***H. pylori* infection**							0.0003
Never		132	39.4		178	53.3	
Ever		203	60.6		156	47.7	
**Tumor position**							
Cardia		78	23.3				
Non-cardia		257	76.7				
**TNM stage**							
I or II		96	28.7				
III or IV		239	71.3				

a χ^2^ test for distribution between the cases and controls.

### Association of rs4072037, rs13361707 and rs2274223 with risk of GC

The genotype distributions of these variants among the controls were all consistent with Hardy–Weinberg equilibrium (*P* = 0.242 for rs4072037, *P* = 0.985 for rs13361707 and *P* = 0.184 for rs2274223). The genotype and allele frequencies of the three SNPs among the cases and controls and the association with risk of GC are shown in Table **2**. Individuals with each variant allele of the three SNPs were significantly associated with altered risk of GC after adjusting for sex, age, BMI, smoking and drinking status, and *H. pylori* serology at allelic levels (adjusted OR = 0.51, 95% CI: 0.37–0.69 for rs4072037; adjusted OR = 1.46, 95% CI: 1.17–1.82 for r13361707; and adjusted OR = 1.31, 95% CI: 1.00–1.71 for rs2274223). However, under the dominant models in which genotypes with one or two variant alleles were assumed to confer the same disease risk as wide-type genotypes, no significant association was observed between rs2274223 and the risk of GC (adjusted OR = 1.33, 95% CI: 0.97–1.84; *P* = 0.079). Furthermore, subgroup analysis stratified by tumor site was established to evaluate the association of genetic variants of the three SNPs with risk of subtype GC (Table **S1**). Under the dominant models, G carriers of rs4072037 were consistently associated with significantly decreased risk of GC among both of the two subgroups, whereas the rs13361707-C carriers were only associated with a significantly increased risk of non-cardia GC (adjusted OR = 1.74, 95% CI: 1.17–2.57; *P* = 0.006) and the rs2274223-G carriers were only associated with a significantly elevated risk of cardia GC (adjusted OR = 2.28, 95% CI: 1.38–3.80; *P* = 0.001).

**Table 2:** Distribution of genotypes and alleles of the three SNPs (rs4072037, rs13361707 and rs2274223) and their association with GC risk.

**SNPs**	**Genotypes**	**Controls (*n* = 334**)	**Cases (*n* = 335**)	***P* value^a^**	**Adjusted OR^a^(95% CI**)
Rs4072037	AA	220 (65.9)	266 (79.4)		1.00 (ref.)
	AG	98 (29.3)	64 (19.1)	0.001	0.53 (0.37–0.77)
	GG	16 (4.8)	5 (1.5)	0.017	0.28 (0.10–0.80)
	AG+GG	114 (34.1)	69 (20.6)	1.3×10^-4 b^	0.50 (0.35–0.71)
Allele	A	538 (80.5)	596 (89.0)		1.00 (ref.)
	G	130 (19.5)	74 (11.0)	2.0×10^-5 c^	0.51 (0.37–0.69)
Rs13361707	TT	102 (30.5)	71 (21.2)		1.00 (ref.)
	CT	165 (49.4)	167 (49.9)	0.053	1.46 (0.99–2.12)
	CC	67 (20.1)	97 (29.0)	0.001	2.10 (1.34–3.26)
	CT+CC	232 (69.5)	264 (78.8)	0.007^b^	1.64 (1.14–2.34)
Allele	T	369 (55.2)	309 (46.1)		1.00 (ref.)
	C	299 (44.8)	361 (53.9)	0.001^c^	1.46 (1.17–1.82)
Rs2274223	AA	217 (65.0)	197 (58.8)		1.00 (ref.)
	AG	109 (32.6)	122 (36.4)	0.170	1.26 (0.91–1.75)
	GG	8 (2.4)	16 (4.8)	0.062	2.33 (0.96–5.65)
	AG+GG	117 (35.0)	138 (41.2)	0.079 **^b^**	1.33 (0.97–1.84)
Allele	A	543 (81.3)	516 (77.0)		1.00 (ref.)
	G	125 (18.7)	154 (23.0)	0.047 **^c^**	1.31 (1.00–1.71)

a Adjusted for age, sex, BMI, smoking and drinking status, and *H. pylori* serology in logistic regression model.

b for dominant genetic models.

c at allelic levels.

### Joint effects of *H. pylori* seropositivity and variants of rs4072037, rs13361707 and rs2274223 on risk of GC

We aimed to clarify the effects of the potential interactions between variants of the three SNPs and *H. pylori* seropositivity on the risk of GC (Table **3**). The risk for GC was significantly increased in subjects with *H. pylori* infection (OR, 1.75; 95% CI: 1.28–2.40) adjusted for sex, age, BMI, smoking and drinking status (data not shown). Compared with individuals with rs4072037 AG/GG genotypes and *H. pylori* seronegativity, those with AA genotypes and *H. pylori* seronegativity were found to have an OR of 2.46 (95% CI, 1.42–4.27) for GC risk, those with AG/GG genotypes and *H. pylori* seropositivity were associated with an OR of 2.30 (95% CI, 1.23–4.31) and those with AA genotypes and *H. pylori* seropositivity were associated with an OR of 3.95 (95% CI, 2.29–6.79). Similarly, compared with individuals with rs13361707 TT genotypes and *H. pylori* seronegativity, the risk of GC increased among those with TC/CC genotypes and *H. pylori* seronegativity (OR, 1.50; 95% CI, 0.90–2.50), and TT genotypes and *H. pylori* seropositivity (OR, 1.51; 95% CI, 0.81–2.80). The risk was only significantly elevated in the group with TC/CC genotypes and *H. pylori* seropositivity (OR, 2.68; 95% CI, 1.62–4.43). Moreover, compared with individuals with rs2274223 AA genotypes and *H. pylori* seronegativity, individuals with AG/GG genotypes and *H. pylori* seronegativity had an almost unaltered risk of GC (OR, 1.01; 95% CI, 0.63–1.62), whereas the risk of GC increased among those with the AA genotypes and *H. pylori* seropositivity (OR, 1.44; 95% CI, 0.97–2.14), and AG/GG genotypes and *H. pylori* seropositivity (OR, 2.45; 95% CI, 1.55–3.88). Table **3** indicates that the most elevated risk of GC was found in individuals with *H. pylori* seropositivity and risk genotypes for each of the three SNPs.

**Table 3:** Joint effects of *H. pylori* seropositivity and variants of the three SNPs on risk of GC.

***H. pylori* status**	**Genotypes**	**Controls(n = 334**)			**Cases(n = 335**)		**Adjusted OR(95% CI**)**^a^**	***P*_*c*_ value ^a^**
		***n***	**%**		***n***	**%**		
	Rs4072037							
-	AG/GG	63	18.9		23	6.9	1.00 (ref.)	
-	AA	115	34.4		109	32.5	2.46 (1.42–4.27)	0.003
+	AG/GG	51	15.3		46	13.7	2.30 (1.23–4.31)	0.017
+	AA	105	31.4		157	46.9	3.95 (2.29–6.79)	6.5×10^-6^
G × E							1.95 (1.41–2.69)	4.9×10^-5 b^
	Rs13361707							
-	TT	57	17.1		32	9.6	1.00 (ref.)	
-	CT/CC	121	36.2		100	29.9	1.50 (0.90–2.50)	0.157
+	TT	45	13.5		39	11.6	1.51 (0.81–2.80)	0.218
+	CT/CC	111	33.2		164	49.0	2.68 (1.62–4.43)	5.4×10^-4^
G × E							1.95 (1.42–2.69)	3.9×10^-5 b^
	Rs2274223							
-	AA	110	32.9		82	24.5	1.00 (ref.)	
-	AG/GG	68	20.4		50	14.9	1.01 (0.63–1.62)	0.964
+	AA	107	32.0		115	34.3	1.44 (0.97–2.14)	0.105
+	AG/GG	49	14.7		88	26.3	2.45 (1.55–3.88)	3.7×10^-4^
G × E							2.09 (1.41–3.10)	2.5×10^-4 b^

a *P* values corrected by Benjamini and Hochberg’s method and adjusted for age, sex, BMI, smoking and drinking status in logistic regression model.

b *P* values for interactions between the genotypes of the three polymorphisms and *H. pylori* serology in logistic regression model adjusted for age, sex, BMI, smoking and drinking status. G × E: gene × environment.

The modification effect indicates significantly multiplicative interactions between *H. pylori* seropositivity and variants of the three SNPs (OR = 1.95, 95% CI, 1.41–2.69 for rs4072037; OR = 1.95, 95% CI, 1.42–2.69 for rs13361707; and OR = 2.09,95% CI: 1.41–3.10 for rs2274223). Moreover, the testing for interactions remained significant (all *P*_G× E_ < 0.05) for the three SNPs and *H. pylori* serology.

### Joint effect of *H. pylori* seropositivity and combined variants of three SNPs on risk of GC

Considering that the three SNPs were all related to host immune response, we further investigated the joint effect of the combined genotypes of the three SNPs and *H. pylori* infection on the risk of GC. All individuals were further grouped on the basis of numbers of risk genotypes, as follows: (a) low-risk group (0 or 1 risk genotype); (b) medium-risk group (two risk genotypes); and (c) high-risk group (three risk genotypes), as shown in Table **4**. The risk genotypes for GC in this study were AA of rs4072037, CT/CC of rs13361707 and AG/GG of rs2274223. Compared with those in the low-risk group with *H. pylori* seronegativity, individuals in other groups all had an increased risk for GC, but a significant GC risk was found only for those in the high-risk group with *H. pylori* seronegativity (OR = 2.40; 95% CI, 1.26–4.63), medium-risk group with *H. pylori* seropositivity (OR = 2.91; 95% CI, 1.75–4.84), and high-risk group with *H. pylori* seropositivity (OR = 4.70; 95% CI, 2.49–8.86).

**Table 4:** Joint effect of *H. pylori* seropositivity and combined variants of three SNPs on risk of GC.

***H. pylori* status**	**Combined three SNPs ^a^**	**Controls (*n* = 334**)			**Cases (*n* = 335**)		***P*_*c*_ value ^b^**	**Adjusted OR(95% CI**)**^b^**
		**n**	**%**		**n**	**%**		
-	Low-risk group	72	21.6		35	10.4		1.00 (ref.)
-	Medium-risk group	77	23.1		64	19.1	0.086	1.63 (0.96–2.77)
-	High-risk group	29	8.7		33	9.9	0.013	2.40 (1.26–4.63)
+	Low-risk group	60	18		45	13.4	0.194	1.46 (0.83–2.57)
+	Medium-risk group	72	21.6		103	30.7	1.0×10^-4^	2.91 (1.75–4.84)
+	High-risk group	24	7.2		55	16.4	9.0×10^-6^	4.70 (2.49–8.86)

a Low-risk group (0 or 1 risk genotype); medium-risk group (2 risk genotypes); high-risk group (3 risk genotypes); the risk genotypes were AA of rs4072037, CT/CC of rs13361707, and AG/GG of rs2274223.

b *P* values corrected by Benjamini and Hochberg’s method and adjusted for age, sex, BMI, smoking and drinking status in logistic regression model.

### Effect of interactions of *H. pylori* seropositivity and variants of three SNPs on GC risk stratified by TNM stage

We performed a subgroup analysis stratified by TNM tumor stage to evaluate the modifying effects of variants of the three polymorphisms on the association between *H. pylori* serology and risk of GC (Table **5**). Significant interactions were observed between *H. pylori* seropositivity and the three SNPs both to the early (I or II) and late (III or IV) stage GC (all *P*_G× E_ < 0.05). The modifying effects of the variants of rs4072037 and rs2274223 on the risk associated with *H. pylori* seropositivity were more pronounced for stage I or II compared with stage III or IV GC. However, for rs13361707, the modifying effect was not markedly altered between early and late stage GC.

**Table 5:** Joint effects of *H. pylori* seropositivity and variants of three SNPs on GC risk stratified by TNM stage.

***H. pylori* status**	**Genotypes**	**I or II stage**				**III or IV stage**		
		**Ca/Co (96/334**)	***P*_*c*_ value ^a^**	**adjusted OR (95% CI**)**^a^**		**Ca/Co (239/334**)	***P*_*c*_ value ^a^**	**adjusted OR (95% CI**)**^a^**
	rs4072037							
-	AG/GG	4/63		1.00 (ref.)		19/63		1.00 (ref.)
-	AA	31/115	0.037	3.83 (1.28–11.44)		78/115	0.024	2.17 (1.20–3.92)
+	AG/GG	14/51	0.054	3.70 (1.13–12.10)		32/51	0.099	1.95 (0.98–3.86)
+	AA	47/105	0.004	6.75 (2.31–19.76)		110/105	5.1×10^-4^	3.32 (1.85–5.94)
	G × E^b^		0.001	2.22 (1.38–3.57)			0.001	1.85 (1.30–2.62)
	rs13361707							
-	TT	6/57		1.00 (ref.)		26/57		1.00 (ref.)
-	CT/CC	29/121	0.104	2.31 (0.90–5.93)		71/121	0.434	1.31 (0.75–2.28)
+	TT	15/45	0.055	3.02 (1.07–8.51)		24/45	0.805	1.14 (0.57–2.26)
+	CT/CC	46/111	0.008	4.06 (1.62–10.15)		118/111	0.010	2.32 (1.36–3.97)
	G × E^b^		0.007	1.91 (1.19–3.06)			1.7×10^-4^	1.94 (1.38–2.74)
	rs2274223							
-	AA	22/110		1.00 (ref.)		60/110		1.00 (ref.)
-	AG/GG	13/68	0.986	1.01 (0.47–2.15)		37/68	0.965	1.01 (0.60–1.69)
+	AA	32/107	0.244	1.48 (0.79–2.75)		83/107	0.188	1.40 (0.91–2.16)
+	AG/GG	29/49	0.002	3.27 (1.67–6.39)		59/49	0.007	2.16 (1.32–3.56)
	G × E^b^		3.2×10^-4^	2.75 (1.59–4.78)			0.004	1.87 (1.22–2.87)

a *P* values corrected by Benjamini and Hochberg’s method and adjusted for age, sex, BMI, smoking and drinking status, and *H. pylori* serology in logistic regression model. Ca: cases; Co: controls.

b Interactions between the genotypes of the three polymorphisms and *H. pylori* serology in logistic regression model adjusted for age, sex, BMI, smoking and drinking status. G × E: gene × environment.

## Discussion

We confirmed the association of three GWAS-identified SNPs (rs4072037, rs13361707 and rs2274223) with GC susceptibility after adjusting for sex, age, BMI, smoking and drinking status, and *H. pylori* serology in an independent case–control study of 335 GC cases and 334 controls in a Chinese population. The results were consistent with the association in the three GWASs [7–9] and other recently published independent replication studies in China [19–21]. However, these studies all lacked data about *H. pylori* infection, and were not able to adjust for the potential influence of *H. pylori* infection. In addition, our study is believed to be the first independent replication to confirm the association between rs13361707 and the risk of GC. Besides, stratification analysis of tumor location showed genetic variants of rs13361707 were only associated with a significantly increased risk of non-cardia GC, while genetic variants of rs2274223 were specifically associated with a significantly increased risk of cardia GC in the present study.

Another novel finding in our study was the identification of significantly multiplicative interactions between the three SNPs and *H. pylori* infection to the risk of GC. To the best of our knowledge, this is the first association study of GWAS-identified genetic loci for GC risk taking account of *H. pylori* infection. We found that the three inflammation-related SNPs (rs4072037, rs13361707 and rs2274223) in the GWAS synergized with *H. pylori* seropositivity tending toward the development of GC. The demonstration of interactions between the three genetic variants and *H. pylori* infection leads us to conclude that the risk for GC will be greater for those with high-risk genotypes and *H. pylori* seropositivity. Individuals with high-risk genotypes should be more alert to avoid and eradicate *H. pylori* infection. Further stratification was conducted for each polymorphism by TNM tumor stage and we found that the joint effects of *H. pylori* seropositivity and variants of the three SNPs on the risk of GC appeared to be more evident in early-stage GC for rs4072037 and rs2274223 than late-stage GC, but was not markedly altered for rs13361707. This suggests that individuals with the risk genotypes of the two SNPs (rs4072037 and rs2274223) and *H. pylori* infection could be more likely to develop early-stage GC.

In this population of Hubei Province, China, *H. pylori* infection rate was 60.6% among the cases and 47.7% among the controls, and a positive association was identified between *H. pylori* infection and GC risk. *H. pylori* could affect tumorigenesis through many pathways, including gastric epithelial cell proliferation and apoptosis, and the inflammatory response. In the inflammatory response, *H. pylori* is not only able to activate proinflammatory COX enzymes, especially COX2, that catalyze the key steps in formation of inflammatory prostaglandins [22], but also to activate phospholipase A2, an enzyme that catalyzes the formation of the prostaglandin precursor arachidonic acid [23,24]. The inflammatory response induced by *H. pylori* leads to the production of mutagenic substances such as inducible nitric oxide synthase, which induces ROS or reactive nitrogen species that cause DNA damage in the gastric epithelial cells [25,26]. It has been shown that gene polymorphisms of several inflammatory factors, such as interleukin (IL)-1β [15], tumor necrosis factor (TNF)-α and IL-10 [16], affect levels of protein expression and are associated with an enhanced risk of developing hypochlorhydria, gastric atrophy, and gastric adenocarcinoma related to *H. pylori*. Besides, many studies have reported that gene polymorphisms, including cytokine or chemokine genes (e.g., IL-1β, TNF-α, IL-10 and IL-8) and the innate immune response genes (e.g., Toll-like receptor and mannose-binding lectin2) responded to *H. pylori* infection, are associated with GC susceptibility [27–30].

Gene-environment interactions are complex, therefore, the exact mechanism regulating the interactions between the three genetic variants and *H. pylori* infection is still unclear. Some studies have shown that the three GWAS-identified polymorphisms at 1q22, 5p13 and 10q23 are all related to genes that are important in the host immune responses. First, rs4072037 in MUC1 is known to determine a splicing acceptor site in the signal peptide region [31], and has an effect on the MUC1 promoter activity in the gastric epithelium [10]. Moreover, MUC1 is a receptor for *H. pylori* [32,33] and provides a protective barrier, which limits both acute and chronic colonization by *H. pylori*, as well as limiting the inflammation induced by *H. pylori* infection [33]. Other findings have shown that MUC1 allele length is associated with susceptibility to *H. pylori* gastritis and GC [34,35], and mice deficient in MUC1 are more susceptible to *H. pylori* gastritis [33]. Second, the SNP rs13361707 is located in the first intron of PRKAA1 (encoding protein kinase, AMP-activated, α 1 catalytic subunit) at 5p13.1. Several SNPs across the 5p13.1 region are associated with GC susceptibility and the peak signals occurred at the rs13361707 index SNP in the GWAS [9]. Libioulle et al. [36] have found that genetic variants in the 5p13.1 region are close to the PTGER4 gene, located 270 kb away in the direction of the centromere, and they correlate with quantitative expression levels of PTGER4. The PTGER4 protein, one of the G-protein-coupled receptor subtypes of PGE2, inhibits growth of human gastric carcinoma cell lines [37] and plays an important role in the inflammatory response caused by *H. pylori*. Third, rs2274223 is a substitution of His to Arg in PLCE1. Some studies have shown that PLCE1 plays a major role in inflammation responses during skin [38] and intestinal [13] carcinogenesis, and PLCE1-deficient mice exhibit marked attenuation of tumor-associated inflammation. Besides, Wang et al. [20] have shown that genetic variants of rs2274223 influenced the expression levels of PLCE1 mRNA in the control specimens. Although these findings provide some supportive evidence of the interactions between the three SNPs and *H. pylori* infection, the detailed mechanism involved is still unknown. Further in-depth molecular studies will be needed to explore more functions of the three GWAS-identified SNPs in the etiology of *H. pylori*-related GC.

Several limitations of our study need to be addressed. First, the sample size, especially the cardia GC cases, was not large and the statistical power of the study may have been limited. However, using a power software R 2.15.2 and epicalc package, we found that the statistical power for the interactions between the three SNPs and *H. pylori* to the risk of GC was above 90%. Second, the accuracy of *H. pylori* serology was limited because specific IgG antibody to *H. pylori* infection in the course of gastric carcinogenesis could have been reduced or degraded in the serum, and gastric biopsy for isolation of *H. pylori* strains was not available. Third, other risk factors such as diet profile and socioeconomic status, as well as the quantity of the smoking and drinking alcohol consumption were not available, therefore, it is difficult for us to adjust the potential confounding bias from them. Finally, the present study was a hospital-based case-control study, which might have resulted in potential selection bias.

In conclusion, the current study is believed to be the first to provide evidence that *H. pylori* infection may interact with the three inflammation-related SNPs (rs4072037, rs13361707 and rs2274223) in the GWASs to contribute to the risk of GC. Our findings emphasize the importance of gene-environment interactions when investigating the etiology of GC. Further studies with different ethnic populations and larger sample sizes and functional assays are warranted to validate these findings.

## Supporting Information

Table S1 Association of the three SNPs (rs4072037, rs13361707 and rs2274223) with GC risk stratified by tumor site.
(DOC)
