# Supplementary material for: Helicobacter pylori Infection Synergizes with Three Inflammation-Related Genetic Variants in the GWASs to Increase Risk of Gastric Cancer in a Chinese Population
Source: PLoS One. 2013 Sep 19;8(9):e74976. doi: 10.1371/journal.pone.0074976 (PMC3777913; doi:10.1371/journal.pone.0074976)
Supplement: Table S1 — Association of the three SNPs (rs4072037, rs13361707 and rs2274223) with GC risk stratified by tumor site. (DOC) [file pone.0074976.s001.doc]

**Table S1.** Association of the three SNPs (**rs4072037,** rs13361707 **and rs2274223)** with GC risk stratified by tumor site

| **Genotypes** | **Cardia GC** | | |  | **Non-cardia GC** | | |
| --- | --- | --- | --- | --- | --- | --- | --- |
|  | **Ca/Co (78/334)** | ***P***  **value ^a^** | **Adjusted OR**  **(95% CI) ^a^** |  | **Ca/Co (257/334)** | ***P***  **value ^a^** | **Adjusted OR**  **(95% CI) ^a^** |
| Rs4072037 |  |  |  |  |  |  |  |
| AA (ref.) | 67/220 |  | 1.00 |  | 199/220 |  | 1.00 |
| AG | 10/98 | 0.151 | 0.22 (0.03-1.73) |  | 54/98 | 0.035 | 0.30 (0.10-0.92) |
| GG | 1/16 | 0.002 | 0.33 (0.16-0.67) |  | 4/16 | 0.012 | 0.61 (0.41-0.90) |
| AG+GG **^b^** | 11/114 | 0.001 | 0.31 (0.16-0.62) |  | 58/114 | 0.003 | 0.56 (0.39-0.82) |
| Rs13361707 |  |  |  |  |  |  |  |
| TT (ref.) | 18/102 |  | 1.00 |  | 53/102 |  | 1.00 |
| CT | 37/165 | 0.407 | 1.30 (0.70-2.42) |  | 130/165 | 0.039 | 1.55 (1.02-2.34) |
| CC | 23/67 | 0.057 | 1.96 (0.98-3.93) |  | 74/67 | 0.001 | 2.21 (1.37-3.58) |
| CT+CC **^b^** | 60/232 | 0.174 | 1.50 (0.84-2.67) |  | 204/232 | 0.006 | 1.74 (1.17-2.57) |
| Rs2274223 |  |  |  |  |  |  |  |
| AA (ref.) | 36/217 |  | 1.00 |  | 161/217 |  | 1.00 |
| AG | 36/109 | 0.006 | 2.09 (1.24-3.54) |  | 86/109 | 0.655 | 1.09 (0.76-1.55) |
| GG | 6/8 | 0.007 | 4.83 (1.55-15.06) |  | 10/8 | 0.202 | 1.88 (0.71-4.98) |
| AG+GG **^b^** | 42/117 | 0.001 | 2.28 (1.38-3.80) |  | 96/117 | 0.469 | 1.14 (0.80-1.61) |

^a^Adjusted for age, sex, BMI, smoking and drinking status, and *H. pylori* serology in logistic regression model.

**^b^** for dominant genetic models.
